# Physical fatigue increases neural activation during eyes-closed state: a magnetoencephalography study

**DOI:** 10.1186/s12993-015-0079-3

**Published:** 2015-11-05

**Authors:** Masaaki Tanaka, Akira Ishii, Yasuyoshi Watanabe

**Affiliations:** Department of Physiology, Osaka City University Graduate School of Medicine, 1-4-3 Asahimachi, Abeno-ku, Osaka city, Osaka 545-8585 Japan; RIKEN Center for Life Science Technologies, 6-7-3 Minatojima-minamimachi, Chuo-ku, Kobe city, Hyogo 650-0047 Japan

**Keywords:** Alpha-frequency, Event-related desynchronization (ERD), Magnetoencephalography (MEG), Physical fatigue

## Abstract

**Background:**

Fatigue, defined as difficulty initiating or sustaining voluntary activities, can be classified as physical or mental. In this study, we use magnetoencephalography (MEG) to quantify the effect of physical fatigue on neural activity under the condition of simulated physical load.

**Methods:**

Thirteen healthy right-handed male volunteers participated in this study. The experiment consisted of one fatigue-inducing physical task session performed between two MEG sessions. During the 10-min physical task session, participants performed maximum-effort handgrips with the left hand lasting 1 s every 4 s; during MEG sessions, 3-min recordings were made during the eyes-closed state. MEG data were analyzed using narrow-band adaptive spatial filtering methods.

**Results:**

Alpha-frequency band (8–13 Hz) power in the left postcentral gyrus, precentral gyrus, and middle frontal gyrus (Brodmann’s areas 1, 2, 3, 4, 6, and 46) were decreased after performing the physical fatigue-inducing task.

**Conclusions:**

These results show that performing the physical fatigue-inducing task caused activation of the left sensorimotor and prefrontal areas, manifested as decreased alpha-frequency band power in these brain areas. Our results increase understanding of the neural mechanisms of physical fatigue.

## Background

Fatigue, which can be classified as physical or mental, is defined as difficulty initiating or sustaining voluntary activities [1]. Physical fatigue can be classified as peripheral or central. Central fatigue, which involves decreased motor output from the primary motor cortex, associates with modulations at anatomical sites proximal to nerves which innervate skeletal muscle and is referred to as a progressive decline in the ability to activate muscles voluntarily [2, 3]. Central fatigue has been shown during sustained or repeated maximal or submaximal isometric voluntary contractions of one limb using electrophysiological techniques. It is responsible for 20–25 % of the force loss of physical fatigue [4].

In addition to physical fatigue, changes in neural activities caused by mental fatigue have been previously investigated [5, 6]. Just before and after fatigue-inducing mental task trials, neural activities were evaluated by using magnetoencephalography (MEG). Since combined fMRI and electroencephalography studies showed a negative correlation between alpha-frequency band power and blood oxygen level-dependent (BOLD) signal in the cerebral cortex [7, 8], mental fatigue-inducing task led to suppression of the spontaneous MEG alpha-frequency band (8–13 Hz) power [i.e., event-related desynchronization (ERD)] in the cerebral cortex, suggesting activation of the brain [5, 6].

Although these studies provided insight into the neural mechanisms of mental fatigue, the mechanisms related to physical fatigue in the central nervous system remain unknown. The aim of our present study was to use MEG to clarify the effect of physical fatigue on brain activity under the condition of simulated physical load. The MEG data were analyzed using narrow-band adaptive spatial filtering methods and the alpha-frequency band ERD during the eyes-closed state was specifically evaluated based on the results of our previous studies [5, 6, 9–12]. Eye-closing was performed in order to minimize the confounding factors related to information processing.

## Methods

### Participants

Healthy male volunteers were enrolled. According to the Edinburgh handedness inventory [13], all participants were right-handed. Current smokers, participants with a history of mental or brain disorders, and those taking chronic medications that affect the central nervous system were excluded.

### Ethics, consent and permissions

All the participants provided written informed consent before participation. This study was approved by the Ethics Committee of Osaka City University (Approval number: 2853) and was conducted in accordance with the principles of the Declaration of Helsinki.

### Experimental design

**Fig. 1:**
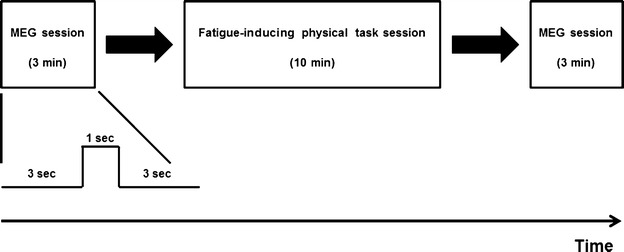
Experimental design. The experiment consisted of one fatigue-inducing physical task session performed between two magnetoencephalography sessions. During the 10-min fatigue-inducing physical task session, participants performed maximum-effort handgrips of 1 s duration every 4 s with the left hand guided by visual and metronome sound cues. Before and after the physical task session, 3-min MEG recordings were obtained during the eyes-closed state

The experiment consisted of one fatigue-inducing physical task session performed between two (before and after) MEG sessions (Fig. 1). During the 10-min fatigue-inducing physical task session participants performed maximum-effort handgrips with the left hand guided by metronome sound cues. During the MEG sessions, 3-min recordings during the eyes-closed state were obtained. Just before and after the fatigue-inducing physical task session, participants were asked to subjectively rate their physical fatigue level using a visual analogue scale (VAS) ranging from 0 (minimum) to 100 (maximum) [14].

### Fatigue-inducing physical task session

The fatigue-inducing maximum-effort handgrips with the left hand were performed using a device (HAND GRIPS 30 kg; IGNIO, Nagoya, Japan). Metronome sounds started 5 min after the beginning of the handgrip trial and were continued until the end of the trial. During the session, the participant watched a fixed mark (+; black mark on a white background) on a screen placed in front of their eyes using a video projector (PG-B10S; SHARP, Osaka, Japan). When a handgrip cue mark (×; black mark on white background) was presented instead of the fixation mark every 4 s, participants were required to perform a handgrip with their left hand at a maximal voluntary contraction level for 1 s by gripping the device. The timing of the visual handgrip cues was the same as that of the metronome handgrip cue sounds. The participants performed repetitive handgrips rather than a continuous handgrip at a maximal voluntary contraction level during the fatigue-inducing physical task session, since the repetitive handgrips could cause more similar levels of physical fatigue than those of the continuous handgrip across the participants.

### MEG session

Each MEG session consisted of 150 blocks, and each block consisted of 3 pacing cues followed by one handgrip cue. During the MEG session, participants heard the pacing sound cues every 1 s with their eyes closed, and then every 4 s, during the handgrip cue period, they were requested to imagine that they were gripping a soft ball with their left hand at a maximal voluntary contraction level for 1 s. The pacing cue consisted of white noise that lasted 33 ms; the handgrip cue consisted of a 1000 Hz tone that lasted 1 s. All the cue sounds were produced by Windows Media Player (Microsoft Corporation, Redmond, WA, USA) and were converted to electric signals by a sound card (Creative X-Fi Audio Processor [WDM]; Creative Technology, Singapore, Singapore) installed in a personal computer (DELL Precision 390; Dell, Round Rock, TX, USA). The sound signals were amplified by an audio amplifier (MA-500U; Onkyo Corporation, Tokyo, Japan) outside of the magnetically shielded room.

This study was conducted in a quiet, temperature-, and humidity-controlled, magnetically shielded room at Osaka City University Hospital. On the day before the study, all the participants refrained from intense mental and physical activities and caffeinated beverages, consumed a normal diet, and maintained normal sleeping hours.

### MEG recordings

MEG recordings were performed using a 160-channel whole-head type MEG system (MEG vision; Yokogawa Electric Corporation, Tokyo, Japan) with a magnetic field resolution of 4 fT/Hz^1/2^ in the white-noise region. The sensor and reference coils were gradiometers 15.5 mm in diameter and 50 mm at baseline, and each pair of sensor coils was separated by a distance of 23 mm. The sampling rate was 1000 Hz with a 200 Hz hard low-pass filter and a 0.3 Hz hard high-pass filter.

### Magnetic resonance imaging overlay

Anatomic MRI was performed using a Philips Achieva 3.0TX (Royal Philips Electronics, Eindhoven, The Netherlands) for all the participants to permit registration of magnetic source locations with their respective anatomic locations. Before MRI scanning, five adhesive markers (Medtronic Surgical Navigation Technologies Inc., Broomfield, CO, USA) were attached to the skin of each participant’s head (the first and second markers were located 10 mm anterior to the left tragus and right tragus, the third 35 mm superior to the nasion, and the fourth and fifth 40 mm to the right and left of the third marker). MEG data were superimposed on MRI scans using information obtained from these markers and MEG localization coils.

### MEG data analyses

As described in detail previously [9–12], MEG signal data were analyzed offline after analogue-to-digital conversion. Magnetic noise originating from outside the shield room was eliminated by subtracting the data obtained from reference coils using a software program (MEG 160; Yokogawa Electric Corporation, Tokyo, Japan), followed by artifact rejection using careful visual inspection. The MEG data were split into segments of 1000 ms in length using a software-trigger. Artifacts were rejected from the signal, cut from the signal, and then 1000 ms segments were created. All the segment data were band-pass filtered at 8–13 Hz by a fast fourier transform using Frequency Trend (Yokogawa Electric Corporation) to obtain alpha-frequency band signals, using the software Brain Rhythmic Analysis for MEG (BRAM; Yokogawa Electric Corporation) [15]. The alpha power analysis was performed before source localization. Localization and intensity of the time–frequency power of cortical activities were estimated using BRAM software, which used narrow-band adaptive spatial filtering methods as an algorithm [16]. The oscillatory power in each voxel was assessed, and the ERD level was calculated as 10 × log_10_[(oscillatory power before the fatigue-inducing physical task)/(oscillatory power after the fatigue-inducing physical task)]. Data were then analyzed using Statistical Parametric Mapping (SPM8, Wellcome Department of Cognitive Neurology, London, UK), implemented in Matlab (Mathworks, Sherbon, MA, USA). The MEG anatomical/spatial parameters used to warp the volumetric data were transformed into the Montreal Neurological Institute (MNI; Montreal, Quebec, Canada) template of T1-weighed images [17] and applied to the MEG data. The anatomically normalized MEG data were filtered with a Gaussian kernel of 20 mm (full-width at half-maximum) in the x, y, and z-axes (voxel dimension was 5.0 × 5.0 × 5.0 mm). The decreased oscillatory power, ERD, for the alpha-frequency band within the time window of 0–1000 ms caused by the fatigue-inducing physical task session was measured on a region-of-interest basis to obtain the neural activation pattern caused by physical fatigue. To enable inferences to be made at a population level, individual data were summarized and incorporated into a random-effect model [18]. The weighted sum of the parameters estimated in the individual analysis was used to create “contrast” images, which were used for group analyses [18]. The resulting set of voxel values for each comparison constituted a statistical parametric map (SPM) of the t statistic (SPM{t}). The SPM{t} was transformed to the units of normal distribution (SPM{Z}). Significant differences in the signal between two eye-closing conditions were assessed using SPM{t} on a voxel-by-voxel basis [18]. The threshold for the SPM{t} of group analyses was set at P < 0.05 (familywise-error corrected for multiple comparisons). The extent threshold in terms of the number of voxels was more than ten voxels. Anatomical localization of significant voxels within each cluster was done using Talairach Demon software [19].

### Statistical analyses

Values are presented as mean ± SD, unless otherwise stated. The paired t-test was used to evaluate differences between two conditions. All P values were two-tailed, and values less than 0.05 were considered statistically significant. Statistical analyses were performed using IBM SPSS 20.0 (IBM, Armonk, NY, USA).

## Results

Of 13 participants, data of 2 participants were excluded from analyses. Because of the MEG noises, it was impossible to analyze the MEG data. In total, 11 healthy male volunteers [age, 22.1 ± 3.1 years (mean ± SD)] were enrolled.

**Fig. 2:**
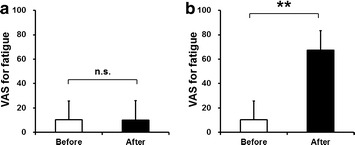
Visual analogue scale values for fatigue of the right and left hands immediately before and after the 10 min fatigue-inducing physical task session. Just before (*open columns*) and after (*closed columns*) the fatigue-inducing physical task session, participants were asked to subjectively rate fatigue levels of the right hand (**a**) and left hand (**b**) using the VAS, ranging from 0 (minimum) to 100 (maximum). Mean and SD. **P < 0.01, paired t-test. *n.s.*, not significant, paired t-test. *VAS* visual analogue scale

To assess the alterations in subjective level of fatigue after the fatigue-inducing physical task session, the changes of VAS score for the right and left hands after the physical session were evaluated. Although the VAS score for the right hand was not altered after the physical task trials (before 10.3 ± 15.6, after 10.0 ± 16.0, P = 0.654, paired t-test), that for the left hand was significantly increased after the physical task trials (before 10.4 ± 15.4, after 67.5 ± 15.8, P < 0.001, paired t-test) (Fig. 2).

**Fig. 3:**
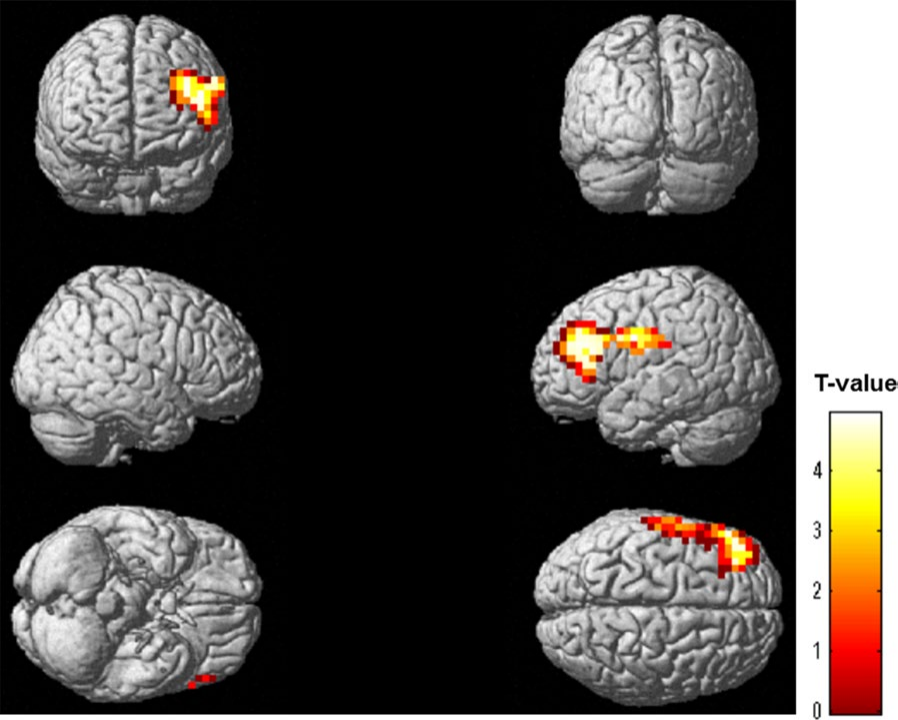
Statistical parametric maps of event-related desynchronization of the alpha-frequency band after the fatigue-inducing physical task session. Statistical parametric maps are superimposed on surface-rendered high-resolution MRIs. Random-effect analyses of 11 participants, P < 0.05, familywise-error corrected for multiple comparisons. *Color bar*: T-values. *MRI* magnetic resonance imaging

To identify the brain regions affected by the physical fatigue, the decreased oscillatory power, as defined by the ERD for the alpha-frequency band, was evaluated after the fatigue-inducing physical task session within the time window of 0–1000 ms. Results are shown in Fig. 3 and Table 1. Among all brain regions, the left postcentral gyrus, precentral gyrus, and middle frontal gyrus [Brodmann’s areas (BA) 1, 2, 3, 4, 6, and 46] showed significant ERDs (random-effect analyses of 11 participants, P < 0.05, familywise-error corrected for multiple comparisons).

**Table 1:** Brain regions that showed event-related desynchronization of alpha-frequency band after the fatigue-inducing physical task session

Locations	Brodmann’s areas	Cluster size	Coordinate (mm)			Z-value
			x	y	z	
Precentral gyrus Middle frontal gyrus Postcentral gyrus	1, 2, 3, 4, 6, 46	28.57	320	−5	28	3.43

*x, y, z* Stereotaxic coordinates of peak of activated clusters

Random-effect analyses of 11 participants (P < 0.05, familywise-error corrected for multiple comparisons)

## Discussion

In this study, we assessed changes of neural activity caused by performing a physical fatigue-inducing task. We showed that a decrease in the alpha-frequency band power in the ipsilateral sensorimotor and prefrontal areas (BA 1, 2, 3, 4, 6, and 46) was induced by physical fatigue. This finding is consistent with the results of our previous studies reporting that a decrease in the alpha-frequency band power assessed using MEG [5, 6] or electroencephalography (EEG) [20] is related to mental fatigue.

Multiple, broadly distributed, and continuously interacting dynamic neural networks are achievable through the synchronization of oscillations at a particular time–frequency band [21]. Combined fMRI and EEG studies showed a negative correlation between alpha-frequency band power and BOLD signal in the cerebral cortex [7, 8]. The alpha-frequency band power is associated with information processing by deactivation of the brain regions [22]. An increase in the alpha-frequency band power reflects the inhibition of cortical information processing, whereas decreased spontaneous alpha-frequency band power (i.e., ERD) reflects the release from inhibition associated with spreading of activation processes [23]. It was shown that a mental fatigue-inducing task led to the suppression of alpha-frequency band power in the cerebral cortex, and this ERD was interpreted as a result of the additional activation of the cerebral cortex in order to cope with the heavy demands of information processing against the fatigue-inducing mental task [5, 6]. Furthermore, alpha band desynchronization associates with attenuation of anticipatory processing related to sensorimotor preparation or ‘readiness to take action’ [24, 25]. Thus, this activation of the cerebral cortex may be a characteristic feature of mental fatigue related to coping with this fatigue.

Similar to the results of studies of mental fatigue, in this study, we showed that a decrease in the alpha-frequency band power in the left postcentral gyrus, left precentral gyrus, and middle frontal gyrus was induced by physical fatigue. This suggests that the ipsilateral sensorimotor and prefrontal areas were activated in order to cope with the heavy demands of physical load during the fatigue-inducing physical task trials. This additional activation of the cerebral cortex may be a characteristic feature of physical fatigue, or rather fatigue in general.

As active muscle fibers become fatigued, we progressively increase voluntary effort to increase the motor output to compensate for central fatigue [26]. Using an electrophysiological technique, existence of this facilitation system was suggested [3]. The facilitation system may include the ipsilateral sensorimotor area: while participants performed a fatigue-inducing physical task, the fMRI activation volume in the ipsilateral sensorimotor area exhibited a steady increase [27]. Since 7–8 % of primary motor cortex neurons are associated with ipsilateral movements [28], additional recruitment of cortical motoneurons from the ipsilateral side may work to compensate for central fatigue.

In addition to the ipsilateral sensorimotor area, activation of the prefrontal area has been shown during physical tasks: EEG signals showed location shifts toward the ipsilateral sensorimotor and prefrontal areas by physical loads [29], and similar activation patterns have been shown in regional cerebral blood flow responses assessed using positron emission tomography, and BOLD responses using fMRI, during the time course of motor tasks [27, 30–32]. Therefore, the ipsilateral prefrontal area may also be involved in the facilitation system, and through activation of the ipsilateral sensorimotor and prefrontal areas, motor output may be maintained until the task requires a maximal effort.

### Limitations

The present study had five limitations. First, the number of the participants was relatively small. To confirm and generalize the results, future studies involving large numbers of participants are essential. Second, we did not examine neural activities during performance of the physical task, because the muscle activity required to press the button can cause electromagnetic noise. Therefore, we focused on the neural activities during the eyes-closed state. Third, electrocortical measures of frequency spectra are modulated by deeper brain structures, and that differential levels of wakefulness and arousal are possible confounders of temporal changes in these data. Fourth, individuals who habitually participate in exercise may have central and peripheral physiological adaptations which may down-regulate subjectively and/or objectively assessed fatigue severity, as well as delay the onset thereof. Finally, although the inclusion of peripheral fatigue indices may have afforded much insight, electromyography activity of skeletal muscle was not assessed.

### Conclusions

In conclusion, we identified changes in oscillatory brain activities due to physical fatigue under the condition of simulated physical load. Physical fatigue may induce activation of certain brain regions that are indirectly related to the motor performance. We believe that our findings are of great value for furthering the understanding of the neural mechanisms of physical fatigue. Our next step is to identify the changes in oscillatory brain activities due to physical fatigue under the condition of actual physical load.

## Abbreviations

MEG: magnetoencephalography
ERD: event-related desynchronization
VAS: visual analogue scale
BRAM: Brain Rhythmic Analysis
SPM: Statistical Parametric Mapping
MNI: Montreal Neurological Institute
BA: Brodmann’s area
EEG: electroencephalography
BOLD: blood oxygen level-dependent
rCBF: regional cerebral blood flow
